# Prior episodic learning and the efficacy of retrieval practice

**DOI:** 10.3758/s13421-021-01236-4

**Published:** 2021-09-20

**Authors:** Mohan W. Gupta, Steven C. Pan, Timothy C. Rickard

**Affiliations:** 1 Department of Psychology, University of California, San Diego, La Jolla, CA 92093-0109 USA; 2 Department of Psychology, University of California, Los Angeles, CA USA; 3 Department of Psychology, National University of Singapore, Singapore, Singapore

**Keywords:** Cued-recall, Testing effect, Retrieval practice, Dual-memory model, Prior knowledge, Individual differences

## Abstract

In three experiments we investigated how the level of study-based, episodic knowledge influences the efficacy of subsequent retrieval practice (testing) as a learning event. Possibilities are that the efficacy of a test, relative to a restudy control, decreases, increases, or is independent of the degree of prior study-based learning. The degree of study-based learning was manipulated by varying the number of item repetitions in the initial study phase between one and eight. Predictions of the dual-memory model of test-enhanced learning for the case of one study-phase repetition were used as a reference. Results support the hypothesis that the advantage of testing over restudy is independent of the degree of prior episodic learning, and they suggest that educators can apply cued-recall testing with the expectation that its efficacy is similar across varying levels of prior content learning. Implications for testing effect theory are discussed.

## Introduction

Extensive research has established that, over a broad range of conditions and populations, some learning methods are more effective than others. Educationally relevant examples include spaced rather than massed practice (Carpenter et al., 2012; Cepeda et al., 2006; Cepeda et al., 2008; Mozer et al., 2009; Rohrer, 2015) and interleaved rather than blocked practice (Foster et al., 2019; Pan et al., 2019; Rohrer, 2012; Rohrer et al., 2020). A third finding in that vein, and the one on which we focus in the current paper, is that retrieval from memory after an initial study trial can produce better learning and retention than does a restudy activity after the initial study trial. For example, answering the question “*what is the powerhouse of the cell?”* yields a higher probability of retrieving “mitochondria” on a later test than does restudying “*the powerhouse of the cell is the mitochondria,*” particularly if correct answer feedback is provided after each test question (Kang et al., 2007; Pashler et al., 2005). That finding has been variously referred to as the retrieval practice effect, test-enhanced learning, and the testing effect.

A typical testing-effect experiment involves two sessions separated by a retention interval ranging from a few minutes to several weeks. The *study* and *training* phases are conducted during session 1 and the *final test* phase is administered during session 2. For the case of cued-recall testing that is explored in the current work, materials such as facts or word-pairs (e.g., lime-salt) are first studied intact. During training, half of the materials are restudied and half are tested (e.g., *lime-?*). In each of the current experiments, correct answer feedback (henceforth, feedback) was provided after each training phase test trial. During the final test in session 2, a cued-recall test is administered for all items. On the final test in such studies, the testing effect (TE) is defined for each participant as the proportion correct for items in the test condition, minus the proportion correct for items in the restudy condition.

The testing effect has been obtained in a variety of contexts (for reviews, see Karpicke et al., 2014; Kornell & Vaughn, 2016; Rickard & Pan, 2018; Roediger & Karpicke, 2006; Rowland, 2014; van den Broek et al., 2016). It has been observed not only in the laboratory, but also in multiple applied settings, including medical resident classroom learning (Larsen et al., 2009), medical skill learning (Kromann et al., 2009), college classrooms using clickers (Lantz, 2010), children’s spelling (Pan et al., 2015), high school classrooms (Nungester & Duchastel, 1982), and university classrooms (McDaniel et al., 2007).

The testing-effect paradigm allows for multiple informative variations. Manipulations explored in the literature include test type (Carpenter & DeLosh, 2005), material type (Kronman et al., 2009; Pan et al., 2015; Roediger & Karpicke, 2006), presence or absence of feedback (Kang et al., 2007), retention interval (Carpenter et al., 2008; Kornell et al., 2011), and blocked versus random sequencing of test and restudy trials during training (Abel & Roediger, 2017), among others. However, one basic aspect of the testing-effect paradigm has rarely been manipulated; namely, the degree of learning that is achieved prior to the training phase. Exploration of that topic should be of value from both theoretical and applied perspectives. For example, in educational contexts, some students are likely to have studied their notes or read a textbook once prior to a quiz, whereas other students may have done so two or more times, presumably yielding increased episodic knowledge of the material prior to the quiz. The question addressed here is whether the effectiveness of that quiz for producing new learning is moderated by that difference in prior, study-based learning.

We investigate that question by manipulating the number of study-phase item repetitions within the cued-recall testing-effect paradigm. We assume that study phase learning will increase monotonically with increasing repetitions. Over three experiments, experimental conditions involved one study phase trial per item (1x study repetition), four study phase trials per item (4x study repetitions), or eight study trials per item (8x study repetitions). Across all experiments, training phase exposure was held constant at one trial per item, in both the test and the restudy conditions. Although there have been a few papers in which the number of study repetitions has been varied over conditions (e.g., Roediger & Karpicke, 2006), there appears to have been no work that directly addresses the effect of increasing the amount of prior study on the testing effect magnitude.

There are three exhaustive possibilities for the effect of study repetition in the current experiments. One hypothesis is that increasing that repetition, while holding training phase exposure constant, will decrease the efficacy of the training phase test relative to restudy (the *attenuation hypothesis*). The dual-memory model of Rickard and Pan (2018), described in the next section, predicts that outcome. An empirical phenomenon that is potentially consistent with the attenuation hypothesis is the pretesting effect (e.g., Richland et al., 2009), in which a test with feedback trial can yield substantially more learning than does a time-equated study trial, even though there is no prior study in either case. The final test performance advantage for pretesting can be as large as or larger than that of the testing-effect paradigm (Pan & Sana, 2021; cf. Latimier et al., 2019). In the current notation, pretesting constitutes the extreme case of 0x study phase repetition.

Alternatively, increased learning through study repetition may not attenuate the effect of a test, but rather enhance it (the *enhancement hypothesis*). That hypothesis might hold, for example, if (1) more study phase repetition yields more learning, as expected, (2) more study phase learning yields a higher proportion correct on the training test, as expected, and (3) learning through that test is greater when the correct answer is retrieved from memory than when it is provided through feedback. In that scenario, the learning advantage of a training phase test versus restudy should be enhanced with increased study phase repetition, all else held constant.

That scenario appears to be predicted by the episodic context theory of retrieval practice (Karpicke et al., 2014). That theory posits that (1) correct retrieval (but not incorrect retrieval) reinstates more of the episodic context that was encoded during an earlier study trial than does restudy, and (2) greater episodic context reinstatement on training test trials is the primary basis for the testing effect. Hence according to that model, the higher the accuracy rate on the training test, the greater the predicted advantage for testing. Furthermore, the degree of episodic context encoding that occurs during the study phase should be an increasing function of the number of study phase repetitions, increasing the upper bound for the degree of context reinstatement that can occur on a training phase test. The episodic context account thus appears to be uniquely consistent with the enhancement hypothesis. Speaking against that possibility, however, is preliminary evidence that learning on a test trial is not causally influenced by retrieval accuracy when there is immediate feedback (Kornell et al., 2015; Rickard, 2020). A third and final possibility is that the amount of study phase learning neither attenuates nor enhances (i.e., is independent of) the efficacy of testing relative to restudy (the *no-effect hypothesis*).

The hypotheses outlined above are best understood in terms of relative underlying memory strengths for restudied and tested items. However, the dependent measure in the current experiments, and in the vast majority of the literature, is proportion correct. More specifically, it is the difference in final test proportion correct in the test and restudy conditions. Therefore, ceiling effects are a potential issue with data interpretation on this topic. For example, as the proportion correct in the restudy condition approaches one, the maximum TE magnitude must drop toward zero. That fact creates a confound if final test restudy proportion correct increases to a high level with increasing study phase repetition and the TE is observed to decrease with increasing study repetition. In that case, it would not be possible, based on the proportion correct measure alone, to differentiate between the hypothesis of process-based attenuation and a mere ceiling effect on the TE.

In the current experiments we used two approaches to address that issue. First, the experimental designs were similar in most respects to those in our prior work on this topic, in which restudy proportion correct on the final test rarely exceeded .5, reducing the possibility of a large ceiling effect confound. Second and more decisively, we used the proportion correct prediction of the dual-memory model of test-enhanced learning (Rickard & Pan, 2018) for the case of 1x study repetition as a reference prediction not just for the 1x study groups of the current experiments – the case for which that model was originally developed to apply – but also for the 4x and 8x study groups. The logic behind that approach is that the dual-memory prediction for the 1x study case has proven highly accurate over multiple 1x study phase repetition cued-recall datasets (Pan & Rickard, 2018; Rickard, 2020). As we elaborate below, if the model prediction for the 1x study repetition case holds for all study repetition conditions in the current experiments, then the no-effect hypothesis will be supported. Alternatively, if the TE magnitude in the 4x and 8x study groups is smaller than that model predicts for the 1x study case, then the attenuation hypothesis will be supported, and if the TE magnitude in those two groups is larger than the model prediction for the 1x case, then the enhancement hypothesis will be supported. That approach does not require direct comparison of observed testing effect magnitudes across different levels of study phase repetition, and thus it circumvents the potential issue of proportion-correct scaling effects.

## The dual memory framework and quantitative model

The dual memory theoretical framework and the corresponding quantitative model is described in detail in Rickard and Pan (2018; see also Rickard, 2020; for application of the model to transfer of test enhanced learning, see Rickard & Pan, 2020). Here we provide a brief description of the framework, specify the quantitative predictions of the model, and summarize the results of prior data fits.

The framework starts with the simple assumption that learning through exact or near exact study repetition – as on a restudy trial in the TE paradigm – has the effect of reactivating and strengthening the *study memory* that was created on the first exposure to an item, wherein strengthening is operationalized as increasing the probability of correct recall at a later time. That is, study creates a single route, or channel, to answer retrieval on a later test, and restudy strengthens that route. We make no further process claims about how strengthening through restudy occurs. Rather, we make a process claim, described next, about how a test trial following a study trial increases the probability of subsequent correct answer recall, relative to a study trial following a restudy trial.

On each training phase test trial, the model assumes two distinct learning events, strengthening of study memory and creation of a separate test memory. Study memory strengthening occurs on both correct test trials and incorrect test trials with feedback. Successful retrieval on the training test must involve reactivation of the study memory that was encoded in the study phase (provided that no pre-experimental associations that would support that retrieval exist), and that reactivation is assumed to strengthen that study memory, just as restudy does. On incorrect initial test trials, the test cue plus the correct answer feedback reconstitute the full studied item, and thus reactivation and strengthening of study memory can occur during feedback, even if the test cue alone was insufficient for that reactivation to occur.

The second learning event on a test with feedback trial is the formation of a separate test memory. We assume that, unlike a restudy trial, a test trial is sufficiently distinct from the initial study trial that a new *test memory* is formed, providing a second and independent route to subsequent answer retrieval. Specifically, on a test trial only the cue is presented, and the presumed task goal is to retrieve the correct response, rather than to study for future retrieval. We take those as sufficient conditions for formation of a separate test memory. More specifically, on a test trial, presentation of the cue word in the context of a goal to retrieve is assumed to create the new memory of that cue presentation event (i.e., cue memory). When the correct response becomes available – either through correct retrieval from study memory or via correct answer feedback – an association is created between that cue memory and that response, yielding what we term episodic *test memory*. Provided that retrieval success through study memory and test memory for tested items on the final test is not highly correlated, those two routes to retrieval provide the basis for explaining the testing effect.

In that theory, the strength of the cue-response association in test memory does not depend on whether the response is correctly retrieved from study memory or provided through feedback; from the “perspective” of the model, only the availability of the correct answer for association with the cue matters, not how that availability occurs. Hence, learning on a test trial with feedback is not causally determined by whether the participant’s retrieved response is correct or incorrect. Assuredly, items with high study memory strength are more likely to support correct retrieval on the training test, and are more likely to be answered correctly on the final test, but in the model that outcome reflects intrinsic differences in associative learning rate across items, not the event of correct versus incorrect retrieval on the training test itself. Hence, the dual-memory model is consistent with the aforementioned studies in which the authors concluded that learning on a test trial with feedback is not causally related to response accuracy (Kornell et al., 2015; Rickard, 2020).

## Quantitative model

The quantitative model was originally designed to apply to the most commonly used cued-recall testing effect design, in which there is one study phase trial for each item and one training phase trial for each item, and where items in the training phase are randomly assigned to be either restudied or tested with feedback. To create that model within the dual-memory framework, several simplifying assumptions were made. Two critical assumptions are that (1) expected study memory strengths are expected to be the same after training for restudied items and tested items with feedback (correct and incorrect), and (2) for tested items, strengths after training are expected to be the same in study memory and test memory. Those memory strengths are expected to different over items, but on average over a larger number of items they are assumed to be the same. However, for each tested item, study and test memory strengths are assumed to be mutually independent (i.e., study memory strength for a particular item after training does not predict test memory strength for that item, and vice versa). Given a few more auxiliary assumptions, Rickard and Pan (2018) showed that, for a hypothetical participant with an infinite number of items randomly assigned to the restudy and test conditions, probability correct in the test condition of the final test (P_T_) is given by the inclusive-or equation:

1 $$ {\mathrm{P}}_{\mathrm{T}}={\mathrm{P}}_{\mathrm{T}-\mathrm{s}}+{\mathrm{P}}_{\mathrm{T}-\mathrm{t}}-{\mathrm{P}}_{\mathrm{T}-\mathrm{s}}{\mathrm{P}}_{\mathrm{T}-\mathrm{t}}, $$

where P_T-s_ is the probability of correct retrieval through study memory for a randomly selected item in the test condition, and P_T-t_ is the probability of correct retrieval through the test memory for a randomly selected item in the test condition. As indicated by the foregoing discussion, P_T-s_ = P_T-t_ in that equation.

It also follows from the model description above that the probability of correct retrieval through study memory in the restudy condition (P_R_) is the same as the probability of correct retrieval through study memory in the test condition (P_T-s_). Hence, in that simplest case quantitative model instantiation, P_T-s_ = P_T-t_ = P_R_. Equation 1 can thus be reduced to

2 $$ {\mathrm{P}}_{\mathrm{T}}={\mathrm{P}}_{\mathrm{R}}+{\mathrm{P}}_{\mathrm{R}}-{\mathrm{P}}_{\mathrm{R}}\ast {\mathrm{P}}_{\mathrm{R}}\kern0.5em =\kern0.5em {2\mathrm{P}}_{\mathrm{R}}-{{\mathrm{P}}_{\mathrm{R}}}^2, $$

and the equation for the TE is

3 $$ \mathrm{TE}={\mathrm{P}}_{\mathrm{T}}\hbox{--} {\mathrm{P}}_{\mathrm{R}}\kern0.5em =\kern0.5em {\mathrm{P}}_{\mathrm{R}}-{{\mathrm{P}}_{\mathrm{R}}}^2 $$

The model therefore predicts that both probability correct in the test condition (P_T_) and the testing effect magnitude are a function solely of probability correct in the restudy condition, P_R_.

For an actual participant in an experiment, the same quadratic equations describe the model’s expected value prediction for the observed proportion correct in the test condition, PC_T_, and for the TE, where PC_R_ is the observed proportion correct in the restudy condition

4 $$ {\mathrm{PC}}_{\mathrm{T}}-\mathrm{predicted}={2\mathrm{PC}}_{\mathrm{R}}-{{\mathrm{PC}}_{\mathrm{R}}}^2 $$

5 $$ \mathrm{TE}-\mathrm{predicted}={\mathrm{PC}}_{\mathrm{R}}-{{\mathrm{PC}}_{\mathrm{R}}}^2 $$

The dual-memory model therefore makes interval-scale proportion correct predictions, separately for each participant, with no free parameters.

For the cued-recall experimental paradigm, with one study trial per item and one training close approximation over datasets from multiple experiments (Rickard & Pan, 2018). Those experiments differed over multiple factors, including the mean restudy proportion correct, the retention interval between training and final test phases (ranging from 1 to 7 days), and materials (paired associates, word-triplets, and history facts).

The model has also provided close fits to the cumulative PC_T_ distribution over participants (Rickard, 2020). That result was demonstrated across the same data sets that are noted above. Because the distribution of proportion correct scores over participants in the restudy and test conditions reflects not just sampling variability but also individual differences (IDs) in task ability (Rickard, 2020; see also the positive correlations between several ID factors and test and restudy proportion correct, reported in Brewer & Unsworth, 2012; Minear et al., 2018; Pan et al., 2015; Robey, 2019), the good distribution fits of Equation 4 in Rickard (2020) indicate that the model accommodates the aggregate effects of ID factors (such as episodic memory ability and intelligence) on overall performance in the testing-effect paradigm. In fact, the model makes a theoretical claim on that point, namely that ID factors can influence overall task performance, but that there are no interactions between ID factors and training task type. Neither the conceptual model development nor the equations that were described above incorporate mechanisms that would allow for such interactions. Thus, according to the model, participants with higher task ability will learn relatively well through both restudy and testing, and participants with lower task ability will learn relatively poorly through both restudy and testing. That prediction follows from Equation 4, which also imposes the stronger constraint that the expected values of PC_T-t,_ PC_T-s,_ PC_R_ are identical for each participant. See Rickard (2020) for further discussion.

The dual-memory model also makes predictions for the case of no feedback on the training test. Following the bifurcation model (e.g., Kornell et al., 2011), the model predicts that there is no task-relevant learning on incorrect test trials with no feedback, in either study memory or test memory. In all other respects, the model for no feedback remains identical to the model described here for feedback. Rickard and Pan (2018) showed through simulation that the model predictions for the case of no feedback are broadly consistent with the empirical observations of (1) a negative testing effect when the retention interval is very short (i.e., a few minutes) but (2) a positive testing effect when the retention interval is longer.

## Predictions of the dual-memory model when there is more than one study phase repetition

As noted earlier, Equation 4 was intended for the case of 1x study per item. In that case, the simplifying assumption in the model that P_R_ = P_T-s_ = P_T-t_ appears to be plausible. However, given that study memory and test memory are assumed to be independent in that model, that equation cannot be mechanistically correct for both the 1x study repetition case and the multiple study phase repetition case (provided that learning increases with study repetition). The assumption is that P_R_ = P_T-s_ remains viable in the multi-study case, because the number of study phase exposures remains the same for items that are assigned to the training phase restudy and test conditions. However, because of the independence assumption, test memory strength, or probability (P_T-t_), would not increase with increasing study repetitions. Rather, P_T-t_ is expected to remain constant as the number of study phase repetitions increases. Hence, the model predicts a different relation between the three probabilities when there are multiple study phase repetitions:

$$ {\mathrm{P}}_{\mathrm{R}}={\mathrm{P}}_{\mathrm{T}-\mathrm{s}}>{\mathrm{P}}_{\mathrm{T}-\mathrm{t}} $$

Holding the value of P_R_ (and hence P_T-s_) constant, it follows from the model equations that the predicted TE will be smaller in the 4x (or 8x) study case than in the 1x study case. Hence the dual-memory model is consistent with the attenuation hypothesis. We will elaborate on this point in the introduction to Experiment 3.

## Testing the three hypotheses

In all three experiments of this paper, we test the attenuation hypothesis against the no effect and enhancement hypotheses. We do so by fitting Equation 4 to all groups, regardless of the number of study phase repetitions, and observing whether for the 4x and 8x study groups the TE is smaller than, equal to, or larger than the prediction of that equation. Based on extensive prior model-fitting outcomes, we expected Equation 4 to fit very well to the data from the 1x study groups of the current experiments, confirming it as an appropriate reference for inference about the 4x and 8x study groups. The design of Experiment 3 will further allow for direct assessment of the mechanistically correct interval scale predictions of the dual-memory model for the 4x study case.

## Experiment 1

In Experiment 1 we used a design with two study repetition groups. The 1x study group received one exposure for each item during the study phase, and the 4x study group received four exposures for each item. The dual memory prediction for the 1x study case (Equation 4) was calculated for each participant in both the 1x and the 4x study groups, using the PC_R_ for each participant in each group to generate the respective PC_T_ predictions. The dual-memory model predicts TE attenuation in the 4x group, such that the observed TE is smaller than predicted by Equation 4 when applied to that group. The enhancement hypothesis, on the other hand, predicts a TE for the 4x group that is larger than predicted by Equation 4. Finally, a finding that Equation 4 fits as well to the 4x study group as it does the 1x study group would be consistent with the no-effect hypothesis, which posits that the efficacy of testing relative to restudy is independent of (i.e., not moderated by) the amount of study phase learning.

### Methods

#### Participants

This experiment was conducted in our laboratory. We recruited 95 student participants from the subject pool at the University of California, San Diego; all completed both sessions. There were 49 participants in the 1x group and 46 participants in the 4x group. Although no power analyses were conducted prior to the current experiments, a power analysis that was conducted for a previous study in our laboratory (see Rickard & Pan, 2020; Experiment 1) indicated that at least 32 participants per group are needed to have power of .8 to detect a difference in proportion correct between two final test conditions of at least .05. In the current studies, the most critical final test comparison is the observed PC_T_ versus that predicted by Equation 4. Participants had a mean age of 20.26 years (2.13).

#### *Materials*, *design, and procedure*

A cued-recall paradigm was used. Materials were 80 English word-pairs. Forty of those pairs were identical to those used by Rickard and Pan (2020). Forty additional pairs were selected from normative data using the same criteria described in Rickard and Pan (2020): nouns that were five to seven letters, one to three syllables, high in concreteness (400–700), frequency of at least 30 per million, and weakly associated. Word pairs were presented horizontally at the center of the computer monitor, with left- or right-side word placement held constant over repetitions. On all test trials, the left-side word was the cue.

Participants were randomly assigned to either the 1x or 4x study group. In session 1, participants first studied all 80 word-pairs for 6 s per trial, either once or four times depending on group assignment. During the immediately following training phase, half of the words were assigned to the restudy condition and half to the cued-recall testing with feedback condition (henceforth, test condition), and those two training tasks were manipulated within-participant. In the final test phase, all words were tested through cued-recall with no feedback. Within each phase, the presentation order of word-pairs or cue-words was randomized independently for each participant. In the training phase, the assignment of word-pairs to restudy or testing was randomized once, and then counterbalanced across participants. In the restudy condition, each word-pair was restudied for 6 s. In the test condition, the cue-word was presented for 5 s, within which time the participants were asked to type the corresponding answer. Both the cue-word and the correct target were then presented for 1 s, constituting feedback. During the training phase in this and subsequent experiments, there was one trial per word-pair in both the restudy and test conditions, yielding 80 total trials, with restudy and test trials randomly mixed. There was a 48-h retention interval between sessions 1 and 2. During session 2, the final test phase was administered, involving one cued-recall test per item with no feedback. The final test was self-paced, so if a participant was unable to recall the target-word, they were allowed to advance to the next cue-word without making a response. The experiment was programmed using E-Prime 2.0, and was run using laboratory desktop computers. All materials are accessible at the Open Science Framework (osf.io/qsrvd).

### Results and discussion

In the training phase, the mean test condition proportions correct were 0.217 and 0.55 in the 1x and 4x groups, respectively, *t*(93) = 3.16, *p* < 0.005, d = 0.46. That result confirms that the study phase repetition manipulation had the expected effect on learning and performance.

**Fig. 1:**
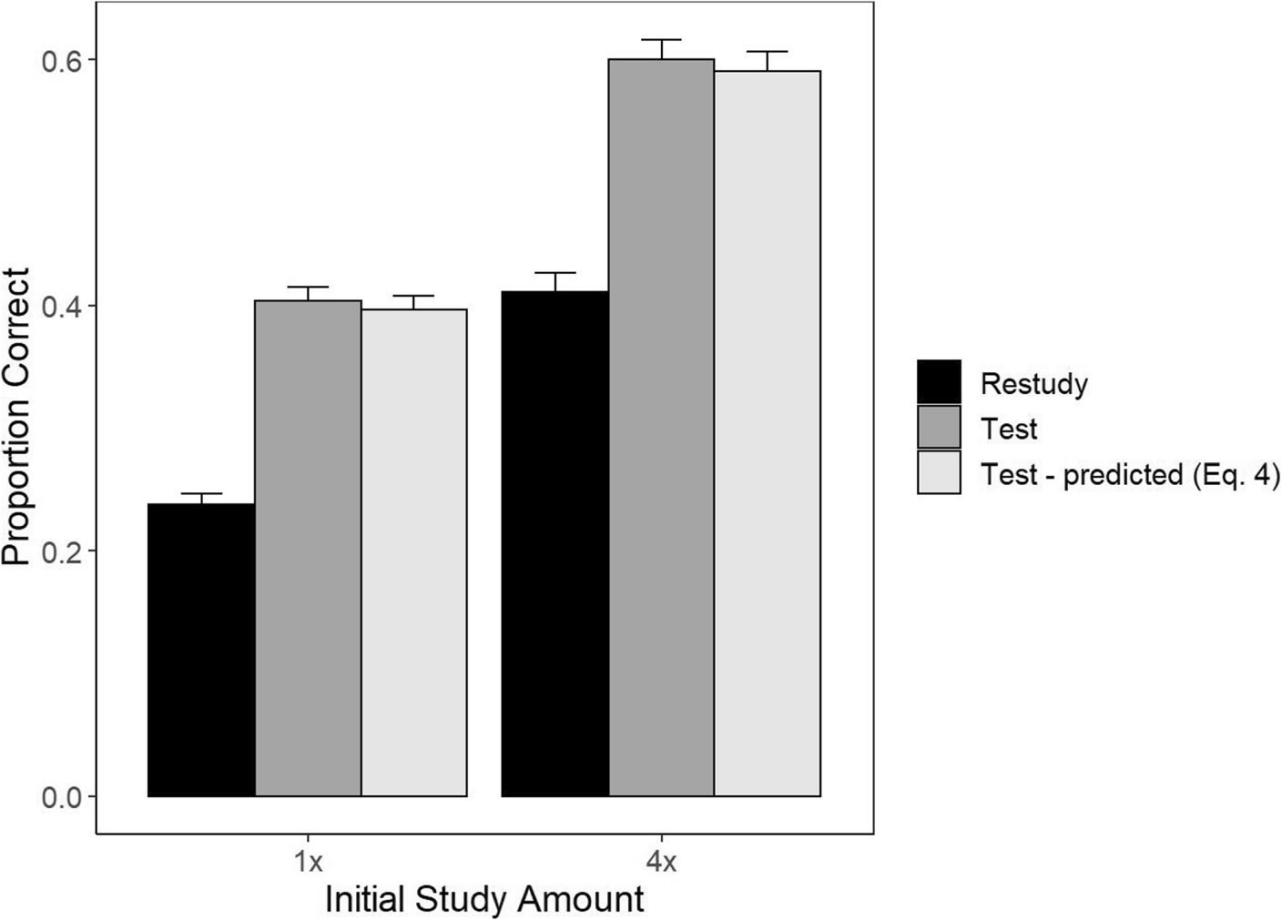
Final test means along with dual-memory model predictions for the test condition in each initial study group. Error bars are standard errors calculated separately for each condition and group

Final test results for proportion correct means, as well as the Equation 4 predictions for the test conditions, are shown in Fig. 1. A mixed-factors ANOVA on the observed proportion correct, with factors of Training Task (restudy vs. test, within participants) and Study Repetition (1x vs. 4x, between), produced a significant (at alpha = .05) main effect of Training Task, *F*(1, 93) = 209.08, *p* < 0.0001, η^2^ = 0.13, and a significant main effect of Study Repetition, *F*(1,93) = 18.1, *p* < 0.0001, η^2^ = 0.12. There was no evidence for an interaction between those two factors, *F*(1,93) = 0.0064, *p* = 0.91, η^2^ = 0.0005. In the restudy condition, mean proportion correct was 0.238 and 0.411 in the 1x and 4x groups, respectively, *t*(93) = 4.07, *p* < 0.0001, d = 0.59. In the test condition, the mean proportion correct was 0.404 and 0.601 in the 1x and 4x groups, respectively, *t*(93) = 4.13, *p* < 0.0001, d = 0.6.

We next evaluated the Equation 4 predictions for the mean proportion correct. A mixed-factors ANOVA with the factors Data Type (observed vs. predicted PC_T_, within; restudy data were excluded from this analysis) and Study Repetition (between; 1x or 4x) yielded a non-significant main effect of Data Type, *F*(1, 93) = 0.51, *p* = 0.48, η^2^ = 0.0003, and a non-significant interaction between Data Type and Study Repetition *F*(1,93) = 0.91, *p* = 0.34, η^2^ = 0.000008. To further evaluate the null hypothesis that the Equation 4 prediction matches the data, a Bayes factor was calculated for both the Study Repetition contrasts. The r (prior) value was set to 0.707 for all tests based on the recommended default Cauchy prior value (Wagenmakers et al., 2018). We used Raftery’s guidelines to interpret the Bayes factor, where 1–3 is weak, 3–20 is positive, 20–150 is strong, and > 150 is very strong evidence for the null hypothesis (Raftery, 1995). The Bayes factor for both the 1x group (*BF*_01_ = 5.97) and the 4x group (*BF*_01_ = 5.16) positively favored the null hypothesis. The Bayes factor for the interaction between Study Repetition and Data Type positively favors the null (*BF*_01_ = 4.52).

**Fig. 2:**
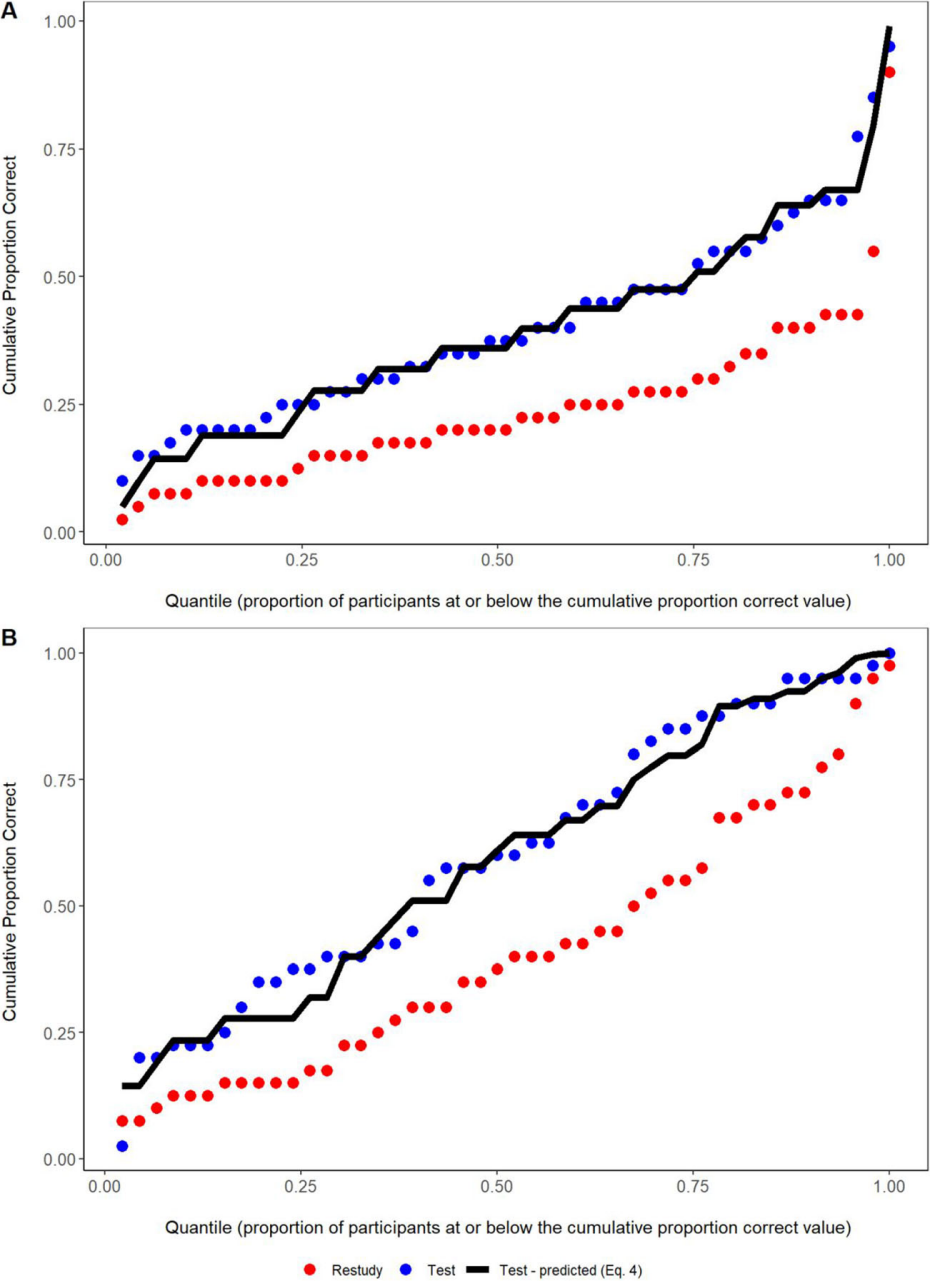
Cumulative distribution plots for both initial study conditions. In both conditions, the model closely predicts the cumulative distribution of the test condition

The cumulative distribution results for PC_T_ are shown in Fig. 2. Equation 4 fitted well overall for both the 1x and 4x study groups, with mean absolute deviations (MADs) between predicted and observed values over quantiles of 0.04 in the 1x study group and 0.047 in the 4x group. Given the relatively small sample sizes for distribution analyses, the modest observed deviations from the predictions are expected even if Equation 4 holds to a close approximation across participants. In Rickard (2020), the model fitted very well to the distribution for a dataset of 509 participants combined over experiments, but exhibited similar localized, idiosyncratic distribution fit deviations when fitted to individual datasets with sample sizes similar to those of the current study.

To conclude, Equation 4 fitted equivalently well to the data from the 1x and 4x study groups. That result is consistent with the no-change hypothesis; it appears that the efficacy of testing with feedback-relative restudy is independent of the amount of prior study-based learning, at least in the current experimental paradigm. That result is inconsistent with the enhancement hypothesis, the attenuation hypothesis, and the dual-memory model extended to the case of 4x study repetition, which predicts testing effect attenuation in the 4x case relative to the Equation 4 prediction.

## Experiment 2

In Experiment 2, which for logistical purposes was conducted online, we explored whether the results of Experiment 1 extend to a higher level of study repetition. One group received 4x study repetitions, replicating the 4x group of Experiment 1, and the other group received 8x study repetitions.

### Methods

#### Participants

Because we conducted this experiment online, we increased sample size as a precaution to help preserve statistical power. We recruited 150 participants from the subject pool at the University of California, San Diego. Thirteen participants did not return for session 2, seven participants erroneously completed only session 2, and one participant was removed because they had previously completed a different experiment with the same stimuli. This left 129 participants whose data were analyzed. Participants had a mean age of 20.69 years (2.38).

#### Materials, design, and procedure

Experiment 2 was identical to Experiment 1, except that the experiment was run online, the number of items was reduced from 80 to 40 (20 in each training phase condition; 20 in each final test condition), and the repetition groups were 4x and 8x. The number of items was reduced to 40 so that participants in the 8x group could complete session 1 in less than 1 h. Participants were randomly assigned into one of two groups of study: 4x or 8x.

To facilitate online data collection, the experiment was coded in JS, HTML, and CSS. The jsPsych library (de Leeuw, 2015) was used for randomization of the training condition and the word-pair order. Participants were emailed a link to session 1. Participants were told they had a 12-h window to complete it. Forty-eight hours after completing session 1, participants were emailed a reminder to perform session 2 and had a 12-h window to do so. No participants were excluded for falling outside of this window. The 4x and 8x groups had average retention intervals of 48.55 (*SD* = 6.20) and 49.39 (12.3) h, respectively; *t*(127) = -0.48, *p* = 0.63.

### Results and discussion

**Fig. 3:**
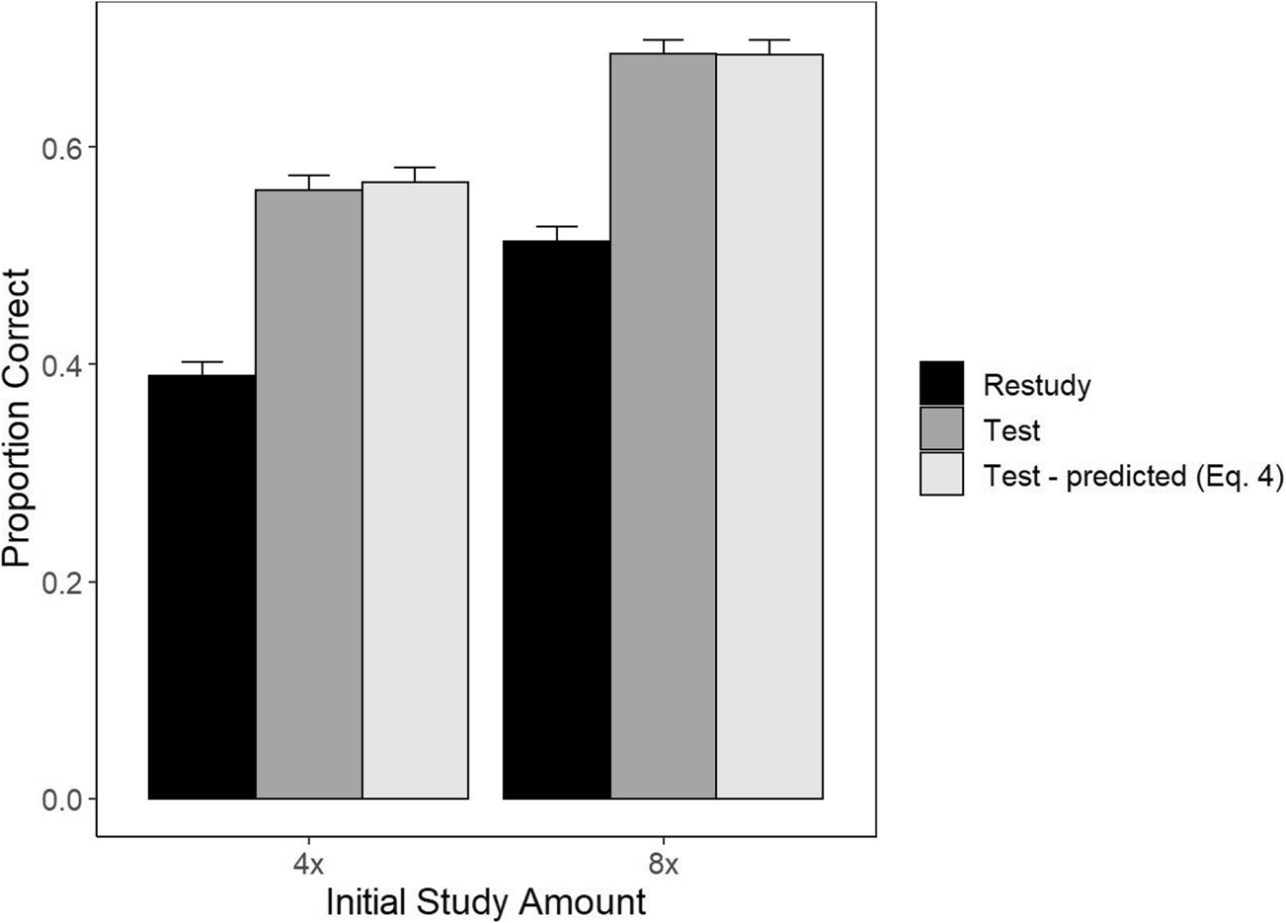
Final test means along with dual-memory model predictions for the test condition in each initial study group. Error bars are standard errors calculated separately for each condition and group

In the training phase, participants had a mean proportion correct on test trials of 0.463 and 0.600 in the 4x and 8x groups, respectively, *t*(127) = -3.16, *p* < 0.005, d = 0.38. Final test results for mean proportion correct are shown in Fig. 3. A mixed-factors ANOVA revealed a main effect of Training Task, *F*(1, 127) = 112.88, *p* < 0.0001, e_x1D702;^2^ = 0.09, and a main effect of Study Repetition, *F*(1,127) = 8.28, *p* < 0.005, η^2^ = 0.05. There was again no interaction between those factors, *F*(1,93) = 0.015, *p* = 0.9, η^2^ = 0.000003. For items that were trained through restudy, participants had a mean proportion correct of 0.389 and 0.513 in the 4x and 8x groups, respectively, *t*(127) = -2.66, *p* < 0.01, d = 0.33. In the final testing phase for items that were trained through recall, participants had a mean proportion correct of 0.560 and 0.685 in the 4x and 8x groups, respectively, *t*(127) = -2.77, *p* < 0.01, d = 0.34.

**Fig. 4:**
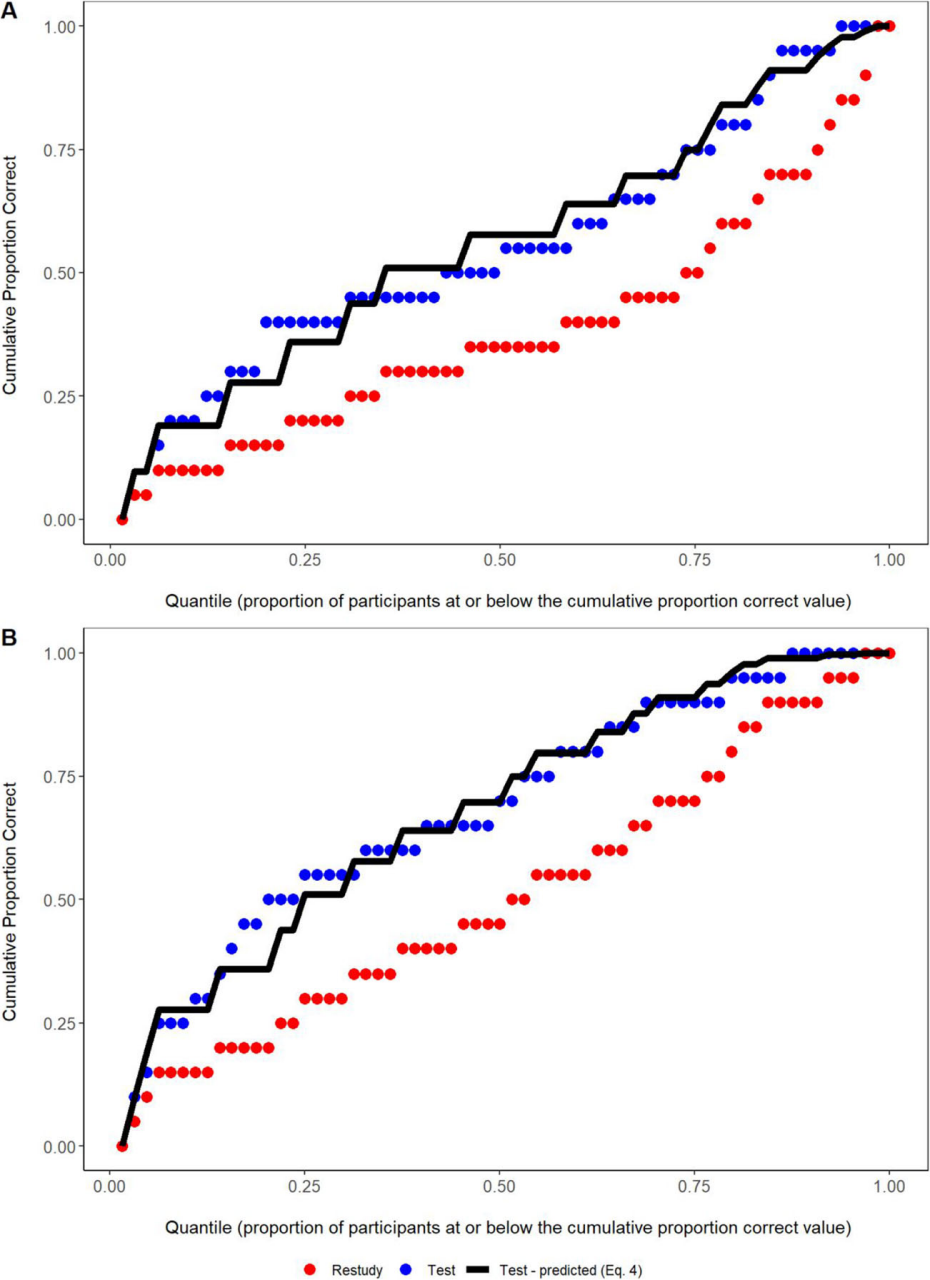
Cumulative distribution plots for both initial study conditions. In both conditions, the model closely predicts the cumulative distribution of the test condition

In the mixed-factors ANOVA for assessing the fit of Equation 4 (identical to that in Experiment 1), there was again a non-significant effect of Data Type *F*(1, 127) = 0.049, *p* = 0.83, η^2^ = 0.00005, and a non-significant interaction, *F*(1, 127) = 0.059, *p* = 0.81, η^2^ = 0.00005. Bayes factor results for Data Type for the 4x group (*BF*_01_ = 6.92) and for the 8x group (*BF*_01_ = 7.3) positively favored the null hypothesis. The Bayes factor for the interaction between Study Repetition and Data Type positively favors null (*BF*_01_ = 6.29). Equation 4 also fitted well to the cumulative distribution, regardless of the number of study repetitions (Fig. 4), with MAD values of 0.035 and 0.027 in the 4x and 8x groups, respectively.

To conclude, results of this experiment again speak against the attenuation and enhancement hypotheses, and in favor of the no-change hypothesis. The results so far support the conclusion that the efficacy of testing relative to restudy is independent of the amount of prior study.

## Experiment 3

In Experiments 1 and 2, study phase repetition was manipulated between participants. In the final experiment, study phase repetition (1x vs. 4x) was manipulated within participants. Hence, the design involved two within-participant factors: training task (restudy vs. testing with feedback) and study phase repetition (1x or 4x). That fully within design was necessary for the mechanistically correct interval scale dual-memory model predictions to be calculated for the 4x repetition (or any multi-repetition) case, as summarized in the next paragraph.

According to the dual-memory model, the proportion correct through test memory (PC_T-t_) is expected to be the same in the 1x and 4x study repetition conditions of the current experiment, given random assignment of items to those conditions. That follows from the assumed independence of study and test memory in the model that was discussed in the *Introduction*. Specifically, because there is only one training phase test trial, PC_T-t_ in both of those conditions is estimated by PC_R_ in the 1x condition (PC_R_1x). In contrast, the estimate of proportion correct through *study memory* for a tested item (PC_T-s_) will depend on the number of study phase repetitions. Specifically, in the 4x study repetition test condition, the estimate of PC_T-s_ is based on the observed PC_R_ in the 4x study condition (PC_R_4x). The mechanistically correct dual-memory prediction equation for PC_T_ in the 4x study phase condition must accommodate those facts. The necessary calculations were illustrated for an example case in the *Introduction*. More formally, for the 4x study condition of the current experiment, the general prediction equation of the model for PC_T_ (Eq. 3)

$$ {\mathrm{PC}}_{\mathrm{T}}={\mathrm{PC}}_{\mathrm{T}-\mathrm{s}}+{\mathrm{PC}}_{\mathrm{T}-\mathrm{t}}-{\mathrm{PC}}_{\mathrm{T}-\mathrm{s}}{\mathrm{PC}}_{\mathrm{T}-\mathrm{t}} $$

cannot be reduced to Equation 4, but can still be expressed entirely in terms of restudy condition proportions correct

6 $$ {\mathrm{PC}}_{\mathrm{T}4\mathrm{x}}=\kern0.5em {\mathrm{PC}}_{\mathrm{R}4\mathrm{x}}+{\mathrm{PC}}_{\mathrm{R}1\mathrm{x}}\kern0.5em \hbox{--} {\mathrm{PC}}_{\mathrm{R}4\mathrm{x}}{\mathrm{PC}}_{\mathrm{R}1\mathrm{x}} $$

In the dual-memory model, calculations are done separately for each participant. Only in the fully within-participant design of Experiment 3 are both PC_R1x_ and PC_R4x_ available for each participant, allowing for calculation of PC_T4x_ in Equation 6.

### Methods

#### Participants

We recruited 67 participants at the University of California, San Diego for this online experiment. Participants were recruited in an identical manner to the preceding experiment and each participant was emailed a link to initiate session 1. Participants were told they had a 12-h window to initiate the experiment. Forty-eight hours after session 1, participants were emailed a reminder to initiate session 2, and they were informed that they had a 12-h window to complete that session and to receive credit. The average retention interval was 45.63 h (9.04).

Fifteen participants did not return for session 2. One participant was removed because they had a retention interval far in excess of 48 h. No other participants were removed from the dataset. This left 51 participants whose data were analyzed. Participants had a mean age of 20.56 years (1.81).

#### Materials, design, and procedure

The design of Experiment 3 was the same as that of Experiment 1, with the exception that all of the conditions were within participant and that the experiment was conducted online (using the same methods as described for Experiment 2). That fully within design yielded four crossed conditions: restudy with 1x study, testing with 1x study, restudy with 4x study, and testing with 4x study. The 80 items from the first experiment were again used, but because all four conditions were within-participant, the intended number of items per condition was 20 instead of 40. There was an error in the program code that randomly assigned items to conditions. Specifically, there was no constraint in the code to assign exactly 20 items to each condition. Over participants, actual item assignment per condition ranged from 13 to 27 items. Over participants, the average number of items assigned per condition was as follows: 1x restudy (20.06), 4x restudy (19.94), 1x tested (19.94), 4x tested (20.06). The programming error was limited to the number of items randomly assigned to each condition during the training phase, and it should not materially affect the results.

### Results and discussion

**Fig. 5:**
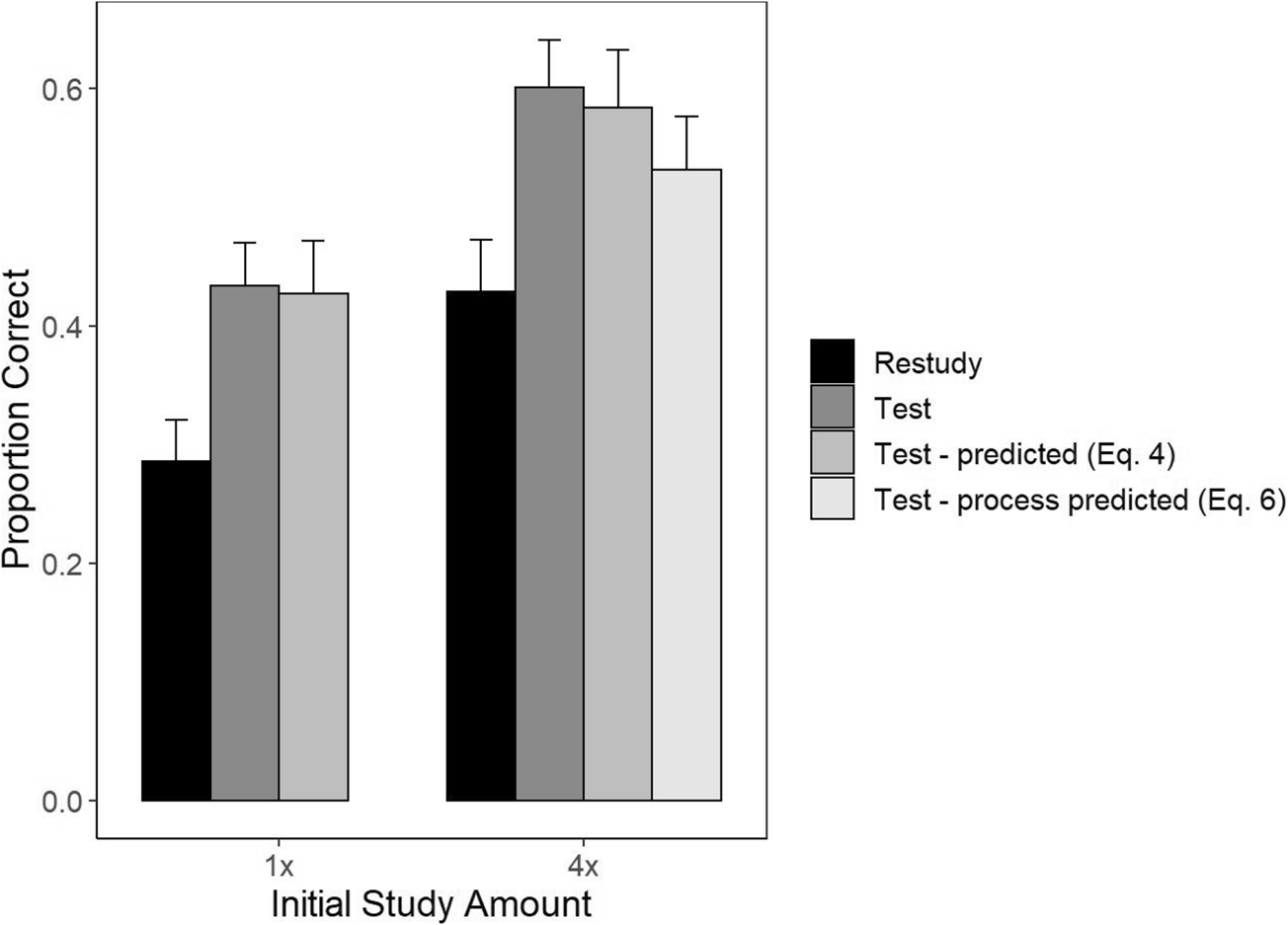
Final test means along with dual-memory model prediction for the test condition in each initial study group and the process model prediction in the 4x study group. Error bars are standard errors calculated separately for each condition and group

In the training phase, the mean proportion correct on test trials was 0.231 and 0.510 in the 1x and 4x conditions, respectively, *t*(50) = 5.73, *p* < 0.0001, d = 1.09. Final test results are summarized in Fig. 5. A repeated-measures ANOVA on the observed proportion correct revealed significant main effects of both Training Task, *F*(1, 50) = 77.64, *p* < 0.0001, η^2^ = .07, and Study Repetition, *F*(1,50) = 63.82, *p* < 0.0001, η^2^ = .07. As for Experiments 1 and 2, there was no significant interaction between those two factors, *F*(1, 50) = 0.65, *p* = 0.42, η^2^ = .0003. Limited to items that were trained through restudy, the mean proportion correct was 0.286 and 0.429 in the 1x and 4x groups, respectively, *t*(50) = 5.76, *p* < 0.001, d = 1.14. Limited to items trained through testing, the mean proportion correct was 0.434 and 0.60 in the 1x and 4x groups, respectively, *t*(50) = 7.37, *p* < 0.0001, d = 1.46.

A repeated-measures ANOVA was also conducted to assess the fit of Equation 4. There were again non-significant effects for both Data Type, *F*(1,50) = 0.42, *p* = 0.52, η^2^ = .00036, and the Data Type by Study Repetition interaction, *F*(1, 50) = 0.087, *p* = 0.77, η^2^ = .000057. The Bayes factors for Data Type in both the 1x condition (*BF*_01_ = 6.26) and for the 4x condition (*BF*_01_ = 5.4) again positively favored the null hypothesis. The Bayes factor for the interaction between Study Repetition and Data Type positively favored the null (*BF*_01_ = 3.38).

Next, we tested the mechanistically correct dual-memory model prediction for PC_T_ in the 4x study condition (using Eq. 6; see the light gray bar to the far right in Fig. 5). That prediction underestimated the observed PC_T_ by 0.068, *t*(50) = -3.02, *p* = 0.01, d = -0.6, whereas Equation 4 had an error of only 0.016, *t*(50) = -0.64, *p* = 0.52, d = -0.13. In summary, the results for mean proportion correct match those of Experiments 1 and 2, supporting the no-effect hypothesis and speaking against the enhancement hypothesis, the attenuation hypothesis, and the quantitative prediction of the dual-memory model that most naturally extends to the multi-study case (Eq. 6).

**Fig. 6:**
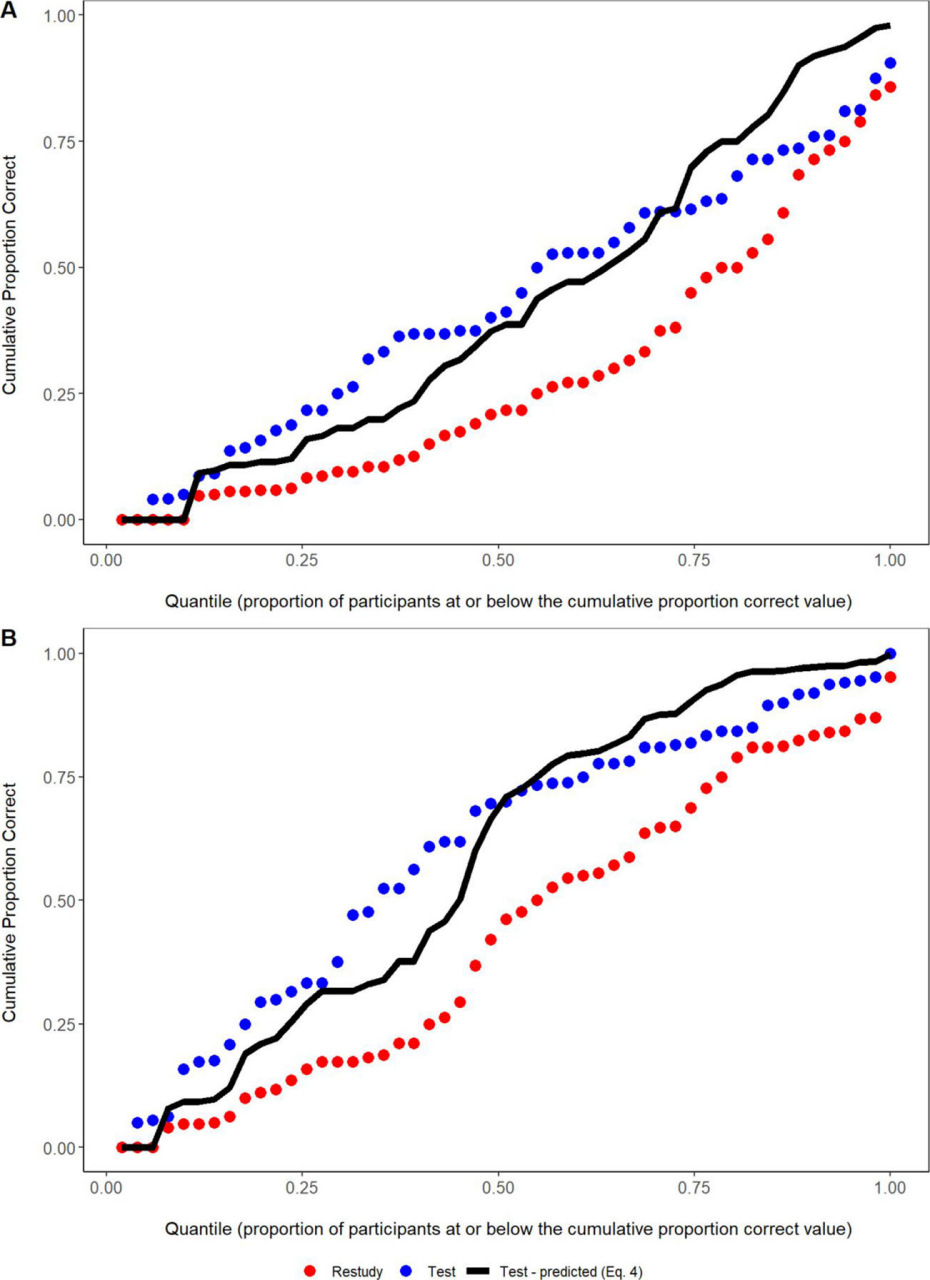
Cumulative distribution plots for both initial study conditions

The cumulative test condition proportions correct, along with the predictions of Equation 4, are shown in Fig. 6, with MAD values of 0.069 and 0.070 in the 1x and 4x conditions, respectively. Those systematically poorer distribution fits to both repetition conditions are surprising in light of the good fits of Equation 4 to the mean proportions correct in all three experiments and to the proportion correct distributions in Experiments 1 and 2 (and also to separate data fits in Rickard & Pan, 2018, and Rickard, 2020). That anomalous result conceivably reflects random factors. Alternatively, it may reflect an unanticipated interaction between the within-participant manipulation of study repetition (i.e., the intermixing 1x and 4x repetition item) and individual differences in task ability, which as noted earlier appear to explain a major component of the proportion correct variability over participants in this paradigm (Rickard, 2020). Participants may have been aware of the uneven number of study repetitions over items and that may have affected learning in a way that interacted with individual differences, although we cannot advance a specific process account. In any case, it appears that, in both study repetition conditions, participants with lower overall task ability (i.e., participants with results on the lower half of the distribution in Fig. 6) benefitted somewhat more in this experiment from testing than Equation 4 predicts and that participants with higher overall task ability (i.e., on the right half of Fig. 6) benefitted somewhat less from testing than Equation 4 predicts.

## General discussion

In this study we explored the effect of prior episodic learning – as manipulated through variation in the number of study-phase item repetitions – on the efficacy of testing. Three hypotheses were considered as the degree of study-based learning increases: attenuation of the testing effect, enhancement of it, and no effect. According to the attenuation hypothesis and its instantiation in the dual-memory model, the efficacy of a test relative to restudy should be a decreasing function of prior study-based learning. Across three experiments, no evidence for that hypothesis was observed. There was also no evidence for the enhancement hypothesis in any of the three experiments. Instead, the results were uniformly consistent with the no-effect hypothesis, according to which the efficacy of a test relative to restudy is unrelated to the amount of prior study.

By using Equation 4, we were able to infer that there was no change in testing efficacy, regardless of the increasing restudy proportion correct, which increased with the amount of study repetitions. That equation has fitted well to the 1x study case across a variety of restudy proportion correct values for both experimental means (Rickard & Pan, 2018) and proportion correct distributions (Rickard, 2020), as well as to the mean proportions correct for the 1x study data from Experiments 1 and 3. Also note that the viability of our conclusions based on application of Equation 4 does not depend on whether the process assumptions of the dual-memory model are correct. For current purposes, Equation 4 is useful for testing the three hypotheses summarized above based solely on the empirical fact that it fits a large corpus of cued-recall testing effect data so well for the 1x study case.

The conclusion that testing effect efficacy is independent of prior study for cued-recall may hold even under circumstances in which proportion correct in the restudy and test conditions approaches one and the testing effect magnitude by that measure approaches zero. There is no principled reason to expect the difference in underlying learning for those two tasks to be reduced when proportion correct reaches the ceiling. Indeed, it is likely that final test proportions correct in that hypothetical experiment could be reduced to virtually any desired values by merely increasing the retention interval, and Equation 4 may still predict the testing effect magnitude across different levels of study phase repetition in that case. Our finding that Equation 4 has held for retention intervals varying between 1 day and 1 week (Rickard, 2020) buttresses that speculation.

Although our results speak to the issue of prior episodic learning and testing efficacy, they do not address the role the prior semantic knowledge has on testing efficacy. Complementary work that addresses that question (e.g., by applying the current methods to evaluate testing for unfamiliar vs. familiar facts, or weakly vs. highly associated word pairs) is warranted.

### Theoretical considerations

The current results appear to rule out the dual-memory model in its current form as an account of the effects of study phase repetition on the TE. One way to adjust that model to account for the current results is to assume that test memory strength is always similar to study memory strength, regardless of the number of prior study phase repetitions. In that account, a stronger study memory promotes a stronger test memory, even though those memories are separate. In that case, both the process model and Equation 4 may still hold regardless of the level of prior study knowledge. That version of the model seems less appealing than the current version, however, because the assumption that study and test memory are fully independent in every sense would no longer hold with respect to the degree of prior study learning, yet would be maintained with respect to the independence of study and test memory strengths for each tested item. The current results also challenge the episodic context theory. As noted earlier, that theory appears to be uniquely consistent with the enhancement hypothesis, given that multiple study phase repetitions were observed to improve training test accuracy, and that those repetitions would be expected to enhance the degree of study phase episodic context encoding.

## Conclusions

The current results make three primary contributions to research on the testing effect. First, they appear to constitute the first direct evidence that the level of prior episodic learning does not substantially moderate the efficacy of testing. That finding remains to be generalized beyond the current experimental design, but it is reasonable to expect it to hold for at least the general case of cued-recall testing. Hence our results suggest that, in applied settings, recall testing can be employed as a potent learning tool across most if not all levels of prior content mastery. Second, Equation 4 now appears to describe the best supported empirical regularity in the testing effect literature, aside from the core finding that testing typically yields more learning at an ordinal level. Even though that regularity will have limits, it is likely in our view to remain a crucible for theory evaluation, particularly in light of the experimental paradigm in which we have observed it, which is minimalist and (we believe) effectively distills the testing effect, excluding most extraneous factors that may moderate outcome. Third, the current results pose challenges to two theories that are under active investigation in the literature: the dual-memory theory and the episodic context theory.
